# Equine Fungal Keratitis due to *Cladorrhinum bulbillosum*

**DOI:** 10.1111/vop.70249

**Published:** 2026-08-17

**Authors:** Abigail Hamilton, Loretta Lacy, Nicole Scherrer

**Affiliations:** ^1^ New Bolton Center University of Pennsylvania School of Veterinary Medicine Kennett Square Pennsylvania USA

**Keywords:** biological scaffold graft, corneal ulceration, cryotherapy, keratomycosis, next‐generation sequencing

## Abstract

**Objective:**

To describe the case details and treatment of an equine fungal keratitis case caused by *Cladorrhinum bulbillosum*.

**Animal Studied:**

A 14‐year‐old Bashkir Curly gelding with a complicated corneal ulcer of the left eye.

**Procedures:**

Corneal cytology was performed, and intraoperative samples were collected for polymerase chain reaction and for aerobic and mycologic cultures. Under general anesthesia, an inferior eyelid subpalpebral lavage system (SPL) was placed to facilitate postoperative therapy. Anterior stromal keratectomy was performed followed by cryotherapy of the keratectomy bed. A biological scaffold graft was secured within the defect and temporary tarsorrhaphy performed to protect the surgical site. Postoperatively, the horse received systemic anti‐inflammatories and topical medications via SPL: antibacterials (ofloxacin, cefazolin), antifungals (voriconazole, luliconazole), and anticollagenase (serum) as well as atropine. Reevaluations were performed every 2–3 weeks until discontinuation of medications.

**Results:**

Following surgical and medical management, the horse showed improvement with resolution of the corneal ulcer over an 8‐week treatment period. Next‐generation sequencing identified *C. bulbillosum* as the predominant organism, and fungal culture supported filamentous fungal infection.

**Conclusion:**

*C. bulbillosum* infection caused equine fungal keratitis in this case. The use of topical voriconazole and luliconazole, combined with surgical intervention (keratectomy, cryotherapy, bio scaffold graft), resulted in a good response. The corneal ulcer healed well with minor scarring over 8 weeks following surgical intervention. To the authors' knowledge, this was the second report of equine fungal keratitis caused by *C. bulbillosum*. This case highlights the utility of next‐generation DNA sequencing and multimodal treatment with adjunctive contemporary therapies.

## 1 Introduction

Corneal ulceration is a common equine ophthalmic disease. A complicated corneal ulcer is one that does not heal in an expected time frame or shows signs of infection [1, 2]. The causes of complicated corneal ulcers include nonhealing ulcers, ulcers caused by persistent trauma, or infected ulcers [3]. Differentiation is frequently achieved through comprehensive ophthalmic examinations and diagnostic sampling such as corneal cytology, culture, and PCR. Infectious causes, including bacterial or fungal, are further investigated to optimize medical treatments with guided antimicrobials or antifungals. Current literature suggests approximately 50% of all equine infectious keratitis cases are attributed to a fungal etiology although this is location dependent [4]. A retrospective study including bacterial and fungal cultures from 39 cases in the Mid‐Atlantic region with severe ulcerative keratitis had 22 positive samples for growth, 18 of which grew a fungus (approximately 46% of the cases) [5].

On ophthalmic examination, equine fungal keratitis (EFK) is often differentiated from other causes of infectious corneal ulcers due to the presence of a white‐yellow plaque, corneal furrow, and reduced vascularization [6]. A recent literature review by Roberts and Gilger suggests that overall, EFK lesions can vary from mild corneal opacity and superficial epithelial defects to deep corneal ulceration with stromal abscessation or fungal plaque formation [4]. Typical lesions are white or multicolored with feathered margins and may resemble “cake frosting” when plaques are present. Furrowing at the lesion edge is commonly associated with fungal infection and more severe disease. As the disease progresses, fungal hyphae can invade deeper into the corneal stroma. The organisms implicated in EFK include *Aspergillus* spp., *Fusarium* spp., *Penicillium* spp., *Microsporum* spp., *Candida* spp., and *Cryptococcus* spp. with *Aspergillus* and *Fusarium* being most common [3, 6]. Fusarium species are more commonly associated with stromal ulceration than *Aspergillus* species, and stromal abscesses may develop when the corneal epithelium heals over an infected wound [3]. There is one previously reported case of EFK caused by *Cladorrhinum bulbillosum*, described in 1966 [7]. Additional reports of *C. bulbillosum* infection have been described in human medicine, including a case associated with ocular trauma from horses' straw bedding [8]. To the authors' knowledge, this is the second report of equine fungal keratitis caused by *C. bulbillosum*. This case highlights the utility of next‐generation DNA sequencing (NGS) for rare pathogen identification as well as the successful use of multimodal medical and surgical therapy with adjunctive contemporary treatments.

## 2 Case Description

### 2.1 Clinical Presentation

Owner consent was obtained for the presentation of patient information and treatment details in this case report. A 14‐year‐old Bashkir Curly gelding first presented in December 2025 for a corneal ulcer of his left eye. The horse was in active use as a lesson horse at a college riding program in Maryland. Routine vaccination and antiparasitic prophylaxis had been maintained. The horse had no reported history of ophthalmic disease. The eye had been managed unsuccessfully on the farm for ~1.5 weeks with neomycin‐polymyxin‐bacitracin, voriconazole and serum four times daily and atropine twice daily. Comfort worsened and referral was recommended and elected.

**FIGURE 1:**
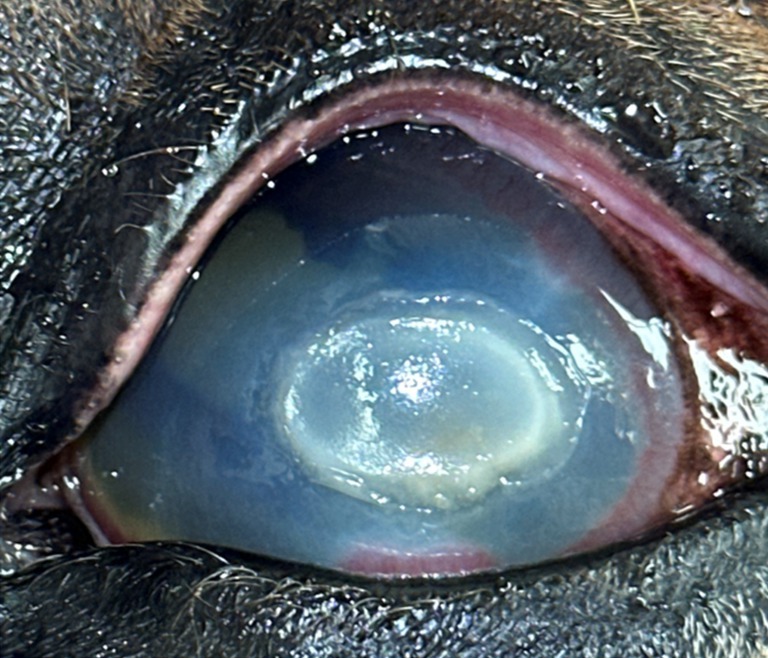
Fourteen‐year‐old Bashkir Curly gelding presented for a complicated corneal ulcer in the left eye.

Ophthalmic examination revealed no significant findings in the right eye. Menace and dazzle responses were present bilaterally, although menace was limited in the left eye. There was severe ocular pain of the left eye (1+ enophthalmos, severe blepharospasm and moderate mucoid ocular discharge). Due to the severity of the corneal disease, the view was severely limited past the cornea. An approximately 15 × 7 mm yellow plaque was apparent along the palpebral fissure with a surrounding furrow and moderate edema with 360‐degree dense limbal vessels extending toward the lesion (Figure 1). A significant decrease in intraocular pressure (24 mmHg OD; 11 mmHg OS) was noted using the TonoVet (iCare). Aqueous flare was graded at 2+, confirming the presence of uveitis. Aqueous flare scoring was performed based on descriptions provided in a semiquantitative preclinical ocular toxicology scoring system (SPOTS) [9]. Fluorescein staining confirmed the presence of the ulcer in the left eye. Based on ophthalmic examination findings, the horse was diagnosed with an infected corneal ulcer and secondary reflex uveitis.

Baseline bloodwork revealed a packed cell volume, total solids, and creatinine within the normal reference intervals. Corneal cytology was performed, which showed many fungal hyphae, numerous inflammatory cells with neutrophils being the most prevalent, and scant epithelial cells. Based on this, a diagnosis of equine fungal keratitis (EFK) was made. Due to the severity of the EFK, surgical treatment under general anesthesia was recommended and elected.

### 2.2 Surgical Treatment and Postoperative Outcomes

For preoperative sedation, the horse was administered 0.02 mg/kg acepromazine with 3 μg/kg detomidine via intravenous (IV) injection. Once in the induction stall, 0.4 mg/kg xylazine was administered via IV injection to improve the plane of sedation. For induction of anesthesia, 2.2 mg/kg ketamine and 0.05 mg/kg midazolam were given IV. For general anesthesia, 10% desflurane in oxygen was administered by inhalation after the horse was intubated, which decreased to 5% desflurane gradually during the procedure. The horse was placed in right lateral recumbency. A continuous rate infusion of 0.5 mg/kg/hour xylazine was utilized for the procedure duration. For local anesthesia and antinociception, phenylephrine and proparacaine drops were administered into the eye. During the surgery, the horse was administered Plasma‐Lyte solution at 5 mL/kg/hour IV. Continuous rate infusion of between 0 and 1 μg/kg/min of dobutamine was utilized to support cardiac contractility when indicated. Invasive blood pressure monitoring was achieved via an 18‐Gauge catheter in the left dorsal metatarsal artery. Electrocardiograph and capnograph were used to monitor heart function and carbon dioxide content in the exhaled air, respectively.

**FIGURE 2:**
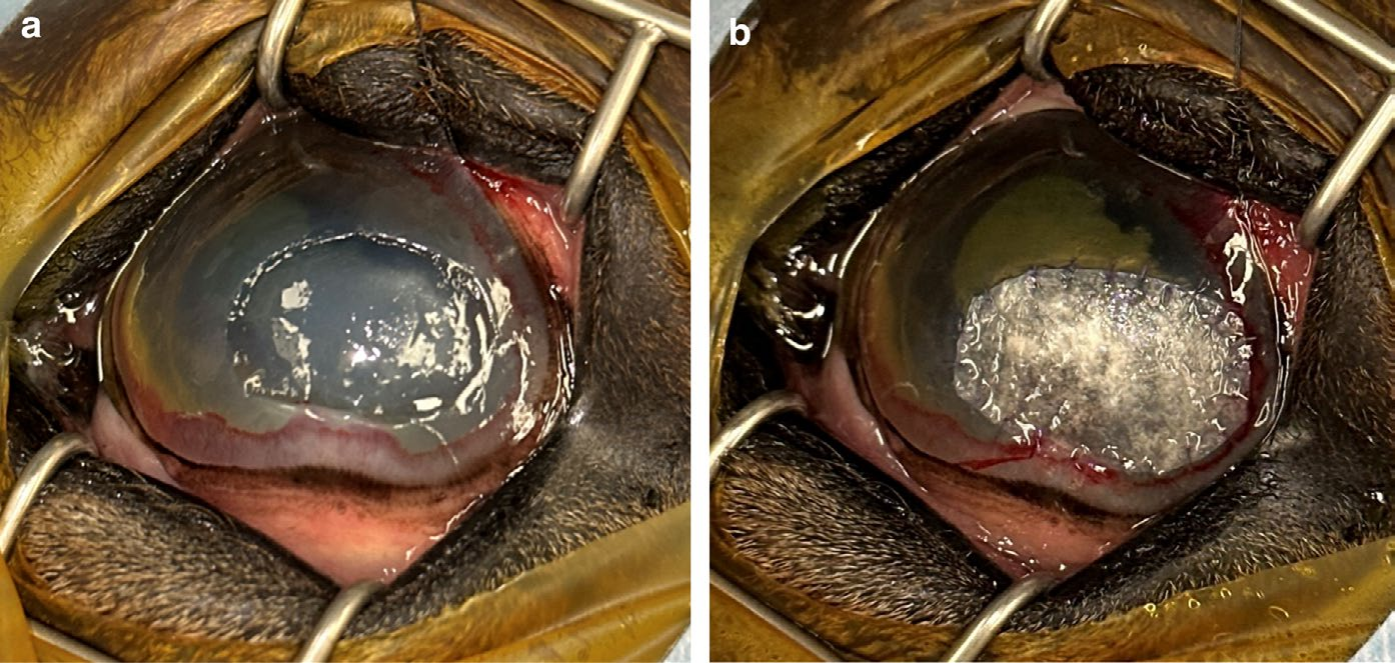
(a) Surgical keratectomy and (b) BioSIS graft placement.

Preoperatively, an inferior eyelid 12 French, 60‐in. MILA subpalpebral lavage (SPL) system with 12 Gauge, straight trocar was placed to facilitate administration of postoperative medications. An anterior stromal keratectomy was performed using a #6900 mini round blade (Beaver‐Visitec) to dissect to a depth of ~40% the corneal thickness followed by the use of a 2.0 mm angled threaded tip crescent blade (Ambler Surgical) to dissect under the plaque (total area removed was ~20 × 15 mm) (Figure 2a). The procedure was performed under magnification (10×) using the Zeiss Lumera 700 ophthalmic microscope. The corneal samples were divided and submitted for NGS (MiDOG All in One Exotic Test), aerobic and mycology cultures. Cryotherapy with the Brymill Cry‐Ac Cryogun using direct contact with a 2‐cm flat probe was performed. Each freeze was ~10 s and was completed when the ice ball extended ~1 mm past the edges of the probe. The total freeze area extended 5 mm past the edges of the keratectomy site. Two freeze–thaw cycles were performed with a 2‐min interval between. A four‐ply BioSIS graft was sized for the keratectomy bed and then sutured within the surgical bed using simple interrupted sutures with Vicryl 8‐0 at 2 mm intervals (Figure 2b). Finally, a temporary tarsorrhaphy was placed using Ethilon 5‐0 suture and rubberband stents to provide protection of the surgical site. The horse recovered routinely from general anesthesia.

Postoperatively, medications were given for analgesia: 20 mg/kg acetaminophen every 12 h orally (PO) and flunixin meglumine every 12 h on a tapering schedule: initially 1 mg/kg PO for 3 days, then 0.5 mg/kg PO for 3 days, finally 0.25 mg/kg PO for 3 days. Topical ophthalmic medications were administered via the SPL system including ofloxacin 0.3%, cefazolin 56 mg/mL, voriconazole 1%, luliconazole 1%, and autologous serum every 4 h and atropine 1% every 12 h. The horse was managed and monitored in the hospital for 2 days post‐procedure and then he was discharged into the owner's care. All medications were continued as previously described following discharge. Throughout the duration of treatment, the SPL remained patent and intact with cautious management and preventative care.

**FIGURE 3:**
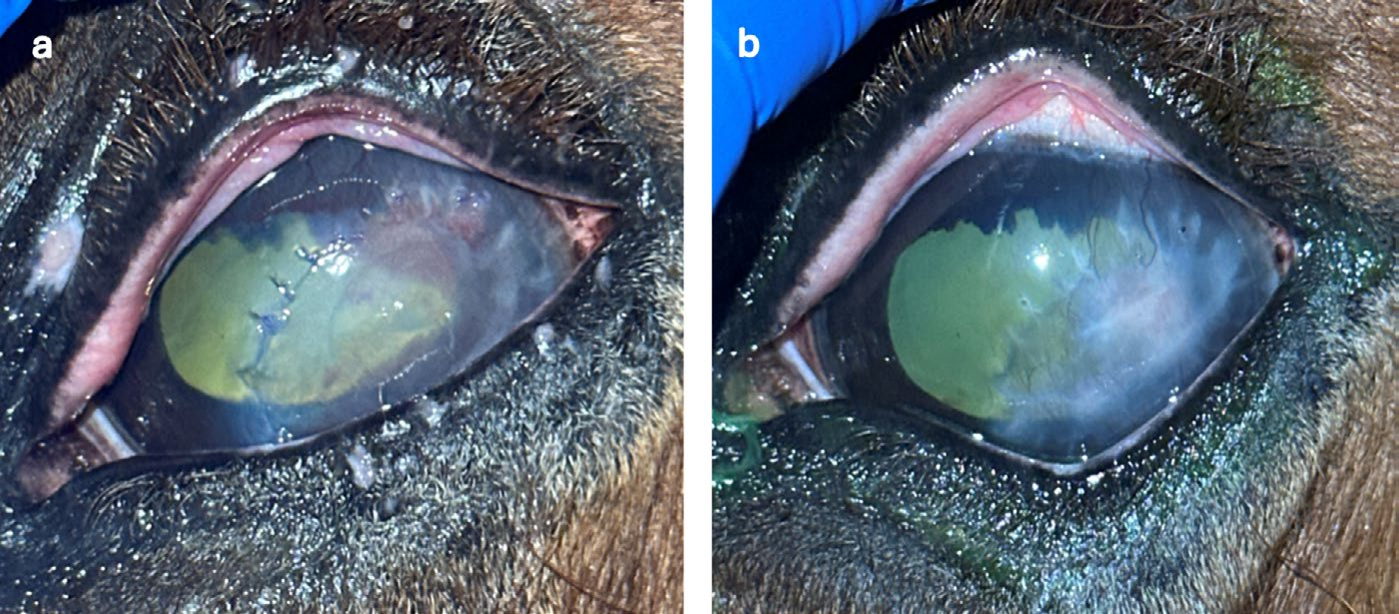
(a) Two‐week follow‐up and (b) 1‐month follow‐up.

The horse re‐presented for initial recheck 2 weeks after discharge (Figure 3a). The temporary tarsorrhaphy was removed, and the ophthalmic examination revealed an improved corneal ulcer of the left eye with an approximately 5 mm ulcer and mild corneal edema remaining centrally within the keratectomy bed and dense vessels approaching from 12 to 6 o'clock. Ocular pain was improved with only mild epiphora. Moderate mydriasis was present. Ocular medications were continued every 4 h via the SPL system, with instructions to discontinue the autologous serum once the provided product ran out. It was advised to continue acetaminophen 20 mg/kg twice daily PO for an additional 2 weeks then discontinue.

**FIGURE 4:**
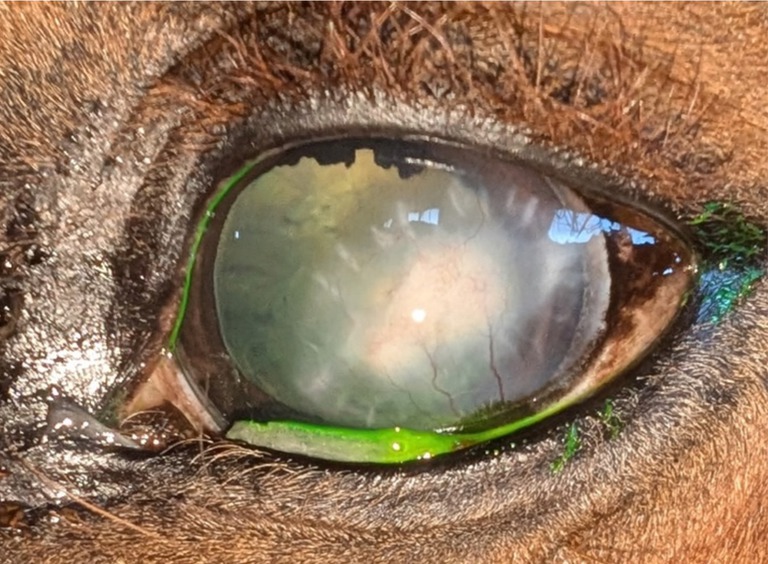
Eight‐week follow‐up and day of SPL removal.

At 5‐weeks postoperative (Figure 3b), comfort was excellent. There were spare vessels associated with the mildly vascularized keratectomy site over the temporal cornea from 2 to 6 o'clock with suture line scars extending from the main opacity. Moderate mydriasis remained present. Following this appointment, topical ophthalmic medications were reduced to every 6 h for 2 weeks, then every 8 h until SPL removal. It was advised to recheck in another 3 weeks with the patient's primary care veterinarian provided no further concerns arose. At the time of the 8‐week recheck appointment, a picture was sent which showed a maximally dilated pupil and a vascularized keratectomy site (Figure 4). SPL removal was approved to conclude treatment.

### 2.3 Next‐Generation Sequencing and Culture Results

NGS with MiDOG Animal Diagnostics results revealed a relative abundance of 90.3% fungi and 9.7% bacteria. The fungus detected was 99.8% *C. bulbillosum*. The bacteria reported were the majority (90.8%) *Streptococceae* species. As described by MiDOG laboratories, NGS is a culture‐independent, DNA‐based diagnostic technique that characterizes the complete microbial population within a sample, including bacteria and fungi. By sequencing all microbial DNA present, NGS enables highly sensitive species‐level identification without requiring target organism selection [10].

Finalized culture report included no bacterial growth on aerobic culture with the presence of filamentous fungus. Mycology culture was concluded as presumptive *Aureobasidium pullulans*. It is likely that the presumptive *A. pullulans* was *C. bulbillosum* based on PCR results and similar phenotypic appearance.

## 3 Discussion

This is the second reported equine fungal keratitis case caused by *C. bulbillosum* with the first being reported approximately 30 years ago. *C. bulbillosum* is a dematiaceous filamentous fungus found in soil where it functions to decompose organic matter [11]. The *Cladorrhinum* genus arises from the Podosporaceae family within the Sordariales order of the Ascomycota phylum [12]. Of the currently 21 recognized species within the genus *Cladorrhinum*, *C. bulbillosum* is the only reported pathogenic strain [13].

In human literature, there are few case reports and case series of fungal keratitis caused by *C. bulbillosum*. The first case of *C. bulbillosum* causing fungal keratitis was reported in 1979 in a 12‐year‐old Argentinian boy related to a piece of straw which penetrated his cornea [8]. A second human case was reported in 2011 involving a farmer who presented with severe pigmented fungal keratitis which did not respond to topical treatment and resulted in eventual perforation of the cornea [13]. The final report of fungal keratitis caused by *C. bulbillosum* was published as a four‐case series of humans in 2020. Three of these four cases were managed successfully with medical therapy alone, and only one required a keratectomy; however, a protracted course of therapy was reported in all of them [14].

Compared to the initial equine report, the case discussed here uses NGS technology for species‐level identification of *C. bulbillosum* and highlights the risk of culture‐based misidentification with similar dematiaceous fungi. NGS as a diagnostic test is performed via parallel sequencing of numerous small fragments of DNA from a sample which are then filtered through a database algorithm to generate a complete report of the microorganisms present in the sample [10]. NGS is used in human medicine for similar applications. A study from 2022 discusses 57 cases where NGS was used and compared to both bacterial and fungal culture results [15]. It was concluded that NGS is useful in nonviral causes of infectious keratitis, especially in cases with negative culture from corneal scrapings. Another paper in human medicine from 2019 reviewed the literature on conventional and advanced methods as applied to the diagnosis of infectious disease of the eye. The conclusion stated “NGS is a novel technology for identifying the pathogens responsible for ocular infections with the potential to improve the accuracy and speed of diagnosis and hastening the selection of the best therapy” [16].

In this case, without the use of NGS, the fungus would have been misidentified through culture as *Aureobasidium pullulans*. Both *C. bulbillosum* and *A. pullulans* often start as pale or pinkish colonies that rapidly turn black as they mature due to melanin production. They also may appear as filamentous fungus, as well as “wet” on certain media due to heavy spore production or polysaccharide secretion [17]. In our case, this misidentification did not alter antifungal choice; however, for other etiologies it may have. NGS would provide a diagnostic advantage in the case of organisms that do not grow well in common culture media. The biggest advantage of NGS is rapid identification of the organisms to guide antifungal therapy choice. Fungal culture is a time‐consuming diagnostic as fungal growth may take up to several weeks which reduces or eliminates the utility for treatment guidance. There is known susceptibility variation between fungal species and antifungal medications chosen may differ depending on results. For example, recent in vitro studies have shown that predominant pathogens such as *Aspergillus* and *Fusarium* exhibit low susceptibility to fluconazole [4]. Fungal susceptibility testing was not performed as it has limited clinical benefit due to the length of time to receive results; in addition, results may be nonspecific [18].

There have been variable findings in signalment, presentation, treatment, and outcome across reported cases of *C. bulbillosum* (Table 1). Despite the species being a pigmented fungus, the case reported here did not present with pigmentation over the ulcerated area. The previously reported equine case similarly did not present with pigmentation. Of the six human cases reported in the literature, three were pigmented [8, 13, 14]. The horse in the current case report did have clinical findings of a furrow surrounding the ulcerated area, a white to yellow, irregular smooth textured plaque, and delayed vascularization in relation to the chronicity of the disease, which all are findings supportive of EFK [5, 19]. However, these clinical signs represent general EFK and have no reported correlation to a specific causative fungal agent [2]. EFK is more prevalent than fungal keratitis in humans. Of equine ulcerative keratitis cases, multiple sources suggest that approximately 30% of these are attributable to underlying fungal causes. This increases to 50% when considering only infectious causes of ulcerative keratitis [20, 21, 22]. Notably, all human cases with histories included were either working with farm animals or had a history of an organic material ocular foreign body. *Cladorrhinum* spp. are found mainly on dung and plant material in soil, which provides a likely explanation for this similarity.

**TABLE 1:** Summary of human cases and single equine case reported with associated treatments and outcomes.

Case	Signalment	Presentation	Antifungal treatment	Medical vs. surgical	Outcome
Zapater and Scattini (1979) [8]	12‐year‐old male following straw in eye working with horses	OD soft, white‐yellowish excrescence, clearly delimited, 4 mm in diameter opacity observed in the center of the cornea.	2% miconazole eye cream, every 2 h for 25 days.	Medical	Healed at 45 days post‐presentation. Patient's eyesight was reduced to a 2/10.
Chopin et al. (1997) [7]	11‐year‐old Percheron cross gelding following ocular penetrating wound	OS cornea cloudy with superficial layers of cornea necrotic and infiltrated with inflammatory cells. Deeper cornea, edematous with increased vascularization.	Topical 3× daily miconazole, reduced frequency gradually over 9 weeks of treatment.	Surgical—keratectomy	Owner reported a small amount of corneal scarring with reported normal vision and horse being used for normal workload.
Gajjar et al. (2011) [13]	42‐year‐old male farmer with liver cirrhosis	OD irregularly marginated 8–9 × 9 mm ulcer with reddish brown pigment in upper half with complete stromal infiltration reaching the endothelium.	Topical natamycin and fluconazole eye drops hourly and atropine 3× daily. Systemic fluconazole (150 mg) once daily.	Medical; Surgery declined	2.5 months examination revealed corneal perforation and a flat anterior chamber. Patient lost to follow‐up.
Natarajan and Mehta (2020) [14]	46‐year‐old male	OD 3.5 × 4 mm epithelial defect with a circular anterior stromal infiltrate, hyphate edges and a 1 mm hypopyon.	Hourly topical natamycin and voriconazole with oral voriconazole tablet 2× daily.	Medical	Healed slowly over 2 months. Epithelial defect healed completely, and scarring took place.
Natarajan and Mehta (2020) [14]	57‐year‐old male following foreign body in eye	OS intact epithelium with a 4 × 3 mm pigmented anterior stromal infiltrate and no hypopyon.	Topical natamycin and voriconazole eye drops hourly with oral tablet ketoconazole 2× daily. No improvement so voriconazole drops changed to 0.15% amphotericin B hourly.	Medical	Healed at 3 months. Follow‐up showed complete ulcer scarring and no recurrence.
Natarajan and Mehta (2020) [14]	48‐year‐old male following fall of vegetative matter in eye	OS intact corneal epithelium with a 4.5 × 4 mm deep stromal infiltrate, 30% corneal thinning, and a deep stromal plaque with no hypopyon.	Topical natamycin and voriconazole eye drops hourly with oral voriconazole 2× daily.	Medical	Healed at 3 months. Complete ulcer scarring with cataract and no recurrence.
Natarajan and Mehta (2020) [14]	50‐year‐old male following foreign body in eye	OD 4.5 × 4.5 mm epithelial defect with corresponding pigmented anterior stromal infiltrate and a 1 mm hypopyon.	Topical natamycin and voriconazole eye drops hourly with oral tablet ketoconazole 2× daily.	Surgical—penetrating keratoplasty with surgical iridectomy	Healed at 6 months from presentation to graft healing. Cataract was revised surgically after healing.

Regardless of the specific etiology, the initial treatment for EFK is similar in human and equine ophthalmology. Surgical anterior stromal keratectomy is elected to remove infected tissue and speed up corneal healing [23]. Graft placement is typically elected if the ulcer is approximately or greater than 50% depth [24]. In cases of fungal keratitis, fungal furrow formation can often continue to worsen secondary to an inflammatory response to aggressive antifungal treatment; for this reason, the author commonly elects placement of an absorbable graft option in cases of EFK to help provide tectonic support. Many consider EFK a surgical disease and there were more cases of success when surgery was performed (85.5% visual) in comparison to medical management alone (78.9% visual) [25]. A retrospective looking at cases where extracellular matrix grafts were used to treat equine infectious ulcerative keratitis saw globe and vision retention in 94% of eyes [25]. The advantage of using an extracellular matrix graft over a conjunctival graft is corneal clarity with most cases having a good to excellent visual outcome. Medical treatment employs a similar approach whether it is completed in isolation or with the addition of anterior stromal keratectomy. A SPL catheter is typically placed in horses due to the duration and frequency of treatment required and lack of patient compliance with ocular medications.

SPL placement was performed routinely prior to surgical intervention in this case to avoid pressure on the globe after the keratectomy was performed or manipulation of the graft. This is the authors' preference. The placement of the SPL in the inferior eyelid is also authors' preference. It has been shown that SPLs placed in the superior eyelid have a higher rate of footplate displacement than those placed in the inferior eyelid [26]. This is especially important since patients are rarely kept in our hospital. For a superior eyelid SPL, a suture break is an emergency to avoid footplate displacement; however, for an inferior SPL, this can be fixed on an elective basis.

General anesthesia was chosen for this patient since we were unable to determine the depth of the lesion preoperatively due to the opaque nature of the fungal plaque. When a deep stromal keratectomy needs to be performed, the surgeon prefers to perform these under general anesthesia to avoid globe rupture. General anesthesia also allows use of higher magnification, via an operating microscope, to help identify abnormal cornea for surgical removal. Standing sedation is often used to perform corneal surgery in the horse to avoid the risks of general anesthesia such as musculoskeletal injury and gastrointestinal disease. The risk of overall complications following elective general anesthesia is relatively low [27]. Although standing sedation does eliminate the risk of musculoskeletal complications, a common postoperative concern is gastrointestinal ileus which is not eliminated with standing sedation [28].

Antifungal selection considers geographical location and aims to eliminate infection while minimizing ocular damage; successfully used agents against EFK include miconazole, natamycin, fluconazole, econazole, voriconazole, clotrimazole, and itraconazole [26]. Voriconazole is frequently used as a first‐line therapy due to its ability to penetrate the intact equine cornea and its broad efficacy against common equine pathogens such as *Fusarium*, *Aspergillus*, and *Candida*. Luliconazole has demonstrated potent in vitro activity against *Aspergillus* and *Fusarium*, with markedly lower minimum inhibitory concentrations and efficacy; however, as evidence remains primarily in vitro, voriconazole continues to be preferred for primary treatment [3]. In this case both antifungals were utilized due to concerns of failure of initial treatment with voriconazole prior to referral. In one of the human case reports, in vitro sensitivity was good for natamycin, amphotericin B, itraconazole, and fluconazole; however, the in vivo response to the medications was poor [13]. Previous cases of *C. bulbillosum* have been medically managed to varying degrees of success using miconazole, natamycin, and/or fluconazole.

Cryotherapy was utilized as an adjunct treatment in this case due to the poorly marginated nature of fungal keratitis. It is often difficult to identify the entirety of the affected tissue and therefore, incomplete removal of fungi is likely. To treat potentially remaining fungi, cryotherapy was utilized throughout the keratectomy site with the goal of destroying any remaining hyphae. This was performed according to recommendations in the literature to achieve an ice crystal 1 mm larger than the probe [29]. Cryotherapy is generally performed in two cycles to maximize destruction of fungal cells while minimizing damage to the surrounding healthy structures. There have been multiple proposed theories as to how this works. The theory most familiar to the authors proposes that the rapid cooling effect leads to the formation of intracellular ice crystals. The crystals disrupt the integrity of the cell membrane and cause denaturation and degradation of proteins within the fungal cells [29]. The subsequent rewarming after the procedure may further compromise the cell membrane and activate the immune system, resulting in the production of interferon by the infected corneal cells [30]. In a rabbit model, the use of cryotherapy shortened the overall treatment time versus topical medications alone [29]. Cryotherapy is not entirely without risks, which are reported to include risk of tissue damage, ocular discomfort and inflammation, limited penetration depth, and variable efficacy. However, with the standardized approach and a controlled environment the authors have found these risks to be minimal. Other adjunct therapies are currently more commonly used in human fungal keratitis but may be considered for similar EFK cases in the future. These include, but are not limited to photodynamic therapy, argon cold plasma, and corneal cross‐linking.

In this novel case of *C. bulbillosum* EFK, a combination of medical and surgical therapy facilitated successful treatment. Anterior lamellar keratectomy with BioSIS graft placement, intraoperative cryotherapy, and temporary tarsorrhaphy surgically addressed the majority of the fungal organisms. The horse was discharged into the care of the owners 2 days postoperatively. This is commonly done at our hospital if owners are capable of medicating at the instructed intervals and closely monitoring blepharospasm and ocular discharge. Even within our hospital, daily ophthalmic examinations are not performed unless clinical signs worsen and suggest that is necessary. Depending on the home situation, horses may need to stay longer in hospital or transition to a rehab facility to receive appropriate care. Vision was maintained with only a mild–moderate scar remaining, which should continue to remodel over the next weeks to months.

## 4 Conclusion

In this study, we report on a case of EFK caused by *C. bulbillosum* that failed initial treatment with voriconazole. EFK was confirmed with a combination of diagnostics: cytology, culture, and PCR. A combination of medical (voriconazole, luliconazole) and surgical management (keratectomy, cryotherapy, four‐ply BioSIS graft) resulted in a successful outcome. The use of NGS should be considered in cases of EFK due to the difficulty in identifying fungal etiology with culture. *C. bulbillosum* should be included as a potential fungal etiology of human and equine infectious keratitis.

## Author Contributions

**Abigail Hamilton:** investigation, writing – original draft, conceptualization. **Loretta Lacy:** writing – review and editing, resources. **Nicole Scherrer:** writing – review and editing, supervision, project administration, resources.

## Funding

The authors have nothing to report.

## Disclosure

The authors have not used AI to generate any part of the manuscript.

## Ethics Statement

All clinical, diagnostic, and therapeutic procedures described in this case report were part of standard veterinary care and were authorized by the horse's owner. Informed consent was obtained from the owner for the use of clinical data and the publication of anonymized case details. Formal institutional ethical approval was not required as the study is a retrospective, observational report of individual clinical care.

## Conflicts of Interest

The authors declare no conflicts of interest.

## Data Availability

Data sharing is not applicable to this article as no new datasets were generated or analyzed during this study.
